# Association of Homocysteine Levels With Medial Temporal Lobe Atrophy Among Carriers and Non-carriers of APOE ε4 in MCI Subjects

**DOI:** 10.3389/fpsyt.2022.823605

**Published:** 2022-04-12

**Authors:** Jun Ma, Ling-Yun Ma, FengYuan Man, Guili Zhang

**Affiliations:** ^1^Department of Radiology, Chuiyangliu Hospital Affiliated to Tsinghua University, Beijing, China; ^2^Department of Neurology, Beijing Tiantan Hospital, Capital Medical University, Beijing, China; ^3^Department of Radiology, PLA Rocket Army Characteristic Medical Center, Beijing, China; ^4^China National Clinical Research Center for Neurological Diseases, Beijing, China

**Keywords:** mild cognitive impairment, clinical subtypes, magnetic resonance imaging, medial temporal lobe atrophy, apolipoprotein E genotype, white matter hyperintensities

## Abstract

**Background:**

Different clinical subtypes of mild cognitive impairment (MCI) involve heterogeneous underlying etiologies. This study investigated the association between demographics, neuropsychological performance, apolipoprotein E (APOE) genotype and magnetic resonance imaging (MRI) measures in patients with MCI (amnestic [aMCI] and non-amnestic [naMCI]).

**Methods:**

This case–control study included 130 aMCI patients, 58 naMCI patients, and 1,106 healthy controls (HCs). APOE genotypes, medial temporal lobe atrophy (MTA), neurological evaluation results, and white matter hyperintensities (WMH) were investigated. Serum folate and vitamin B12 concentrations were analyzed by radioimmunoassay, and plasma hyperhomocysteinemia (Hcy) was assessed by a high-performance liquid chromatography-fluorescence method.

**Results:**

Serum folate levels were significantly lower, but plasma Hcy levels were higher, in patients with aMCI and naMCI than in healthy controls. There were significantly higher MTA scores in the aMCI group than the healthy control group. Multiple linear regression showed that serum Hcy and folate concentrations were positively associated with MTA (*p* < 0.05), while APOE4 showed a significant negative association with MTA in the aMCI group (*p* < 0.01). In addition, moderate/severe WMH showed a significant negative association with MTA in the naMCI and HC groups (*p* < 0.01).

**Conclusion:**

The combined presence of APOE4 and Hcy is associated with aMCI in elderly individuals, while moderate/severe WMH is related to naMCI, which suggests etiological differences across MCI subtypes.

## Introduction

Mild cognitive impairment (MCI) is a clinical syndrome characterized by cognitive deficits and is considered the transitional cognitive state between normal cognitive aging and dementia (1). A substantial proportion of MCI patients will develop dementia (2). Based on the impaired cognitive domains, MCI can be divided into two main subtypes: amnestic MCI (aMCI), in which memory loss is predominant, and non-amnestic MCI (naMCI), in which impairments in other cognitive domains, such as attention, language, visuospatial, and/or executive function (EF), are more significant (3). Preliminary studies have indicated that MCI subtypes may exhibit etiological heterogeneity and distinct brain pathologies (4). While aMCI tends to be characterized by core memory impairment and is regarded as a prodromal form of Alzheimer's disease (AD), naMCI will more likely convert to other forms of dementia, such as vascular dementia or dementia with Lewy bodies (5). Recent studies have suggested that aMCI may represent early AD pathology (6). In contrast, the naMCI subtypes are more strongly associated with cerebrovascular or other diseases (5).

The association between homocysteine (Hcy) levels and dementia risk has been studied for many years. Current evidence suggests that homocysteine has important effects on the human body. Related evidence suggests that homocysteine accelerates endothelial damage as well as brain amyloid deposition (7), which are major pathological processes in dementia (8). Furthermore, it has been proposed that elevated homocysteine levels, which induce neurotoxicity (9), are associated with MCI and AD cortical atrophy and hippocampal atrophy (10, 11). Advances in magnetic resonance imaging (MRI) have allowed quantification of features of the hippocampus that reflect changes in cognition and MCI (12). In addition, the ε4 allele of the apolipoprotein E gene (*APOE*ε4) is one of the most important and well-replicated genetic risk factors for the onset of AD and MCI (13, 14). A series of studies have shown that the presence of the ε4 allele increases the risk of MCI progressing to AD. However, the prevalence of the *APOE* ε4 allele varies substantially among MCI subtypes. These factors may interact differently in distinct MCI subtypes and thus affect the severity of cognitive impairment in distinct MCI subtypes differently.

Previous studies have suggested a significant association between elevated levels of homocysteine and hippocampal atrophy among subjects with MCI and AD, and aMCI has been associated with MTA (15); furthermore, several studies have indicated that MRI can be used to assess MTA and accurately predict aMCI progression to AD.

Therefore, to further investigate the etiological heterogeneity that underlies MCI, this study compared the visual MTA scores, MRI-measured WMH, and apolipoprotein E genotype of patients diagnosed with two MCI subtypes for the first time in a Chinese population. Thus, we aimed to investigate the risk factors for aMCI and naMCI associated with MTA.

## Methods

### Participants

Participants were recruited from the Beihua Hospital Memory Clinic and Department of Neurology between August 2013 and August 2015. The inclusion criteria were as follows: aged 55 years or older and a diagnosis of MCI but not dementia, according to the Diagnostic and Statistical Manual of Mental Disorders Fourth Edition (DSM-IV) (16). Additional requirements included a Clinical Dementia Rating scale score of 0.5. The exclusion criteria comprised cognitive impairment attributed to a somatic, psychiatric, or neurological disorder, such as a neurological infection, cerebrovascular accident, severe depression, alcohol abuse, neurodegenerative diseases such as Parkinson's disease, severe head trauma, or a brain tumor. This research protocol was approved by the Ethics Committee of the Affiliated Hospital of Beihua University. In this study, written informed consent was obtained from all participants or their caregivers. One hundred eighty-eight participants were initially classified into two groups: the aMCI group (*n* = 130) and the naMCI group (*n* = 58). All subjects received a comprehensive neuropsychological evaluation, MRI scan, and blood draws for genotyping.

### Neuropsychological Examination

All subjects performed a battery of neuropsychological tests that assessed five cognitive domains, including memory, language, executive function, attention, and visuospatial function. This neuropsychological battery consisted of the following assessments: episodic memory: immediate or delayed recall from the Auditory Verbal Learning Test (WHO); visuospatial ability: clock drawing test (CDT); language: Boston naming test (BNT); attention: digit span (Trail Making Test Part A); and executive function: Trail Making Test Part B. The Chinese version of the Mini-Mental State Examination (MMSE) was used to assess global cognitive function. The Clinical Dementia Rating (CDR) scale was used to evaluate the severity of cognitive dysfunction. The activities of daily living (ADL) test was used to measure the ability to engage in daily living activities. Raw scores were converted to age-, education-, and sex-corrected z scores according to locally collected normative data or published normative data, and these z scores were used for subsequent analyses.

### MCI Diagnostic Criteria and Classification

The MCI diagnostic criteria were as follows: (1) the patient scored 1.5 standard deviations (SDs) below the average score of healthy control subjects after correction for age, sex, and education on at least one neuropsychological test; (2) the patient did not fulfill the diagnostic criteria for dementia according to the DSM-IV criteria; (3) the patient had an overall CDR score of 0.5; and (4) the patient showed no evidence of impairment in the ADL test.

Each participant was classified as normal or MCI based on five sets of neuropsychological criteria for MCI that differed in their characterization (16) of objective cognitive impairment (17). The participants were classified as having amnestic MCI (aMCI) if the memory domain was impaired or non-amnestic MCI (naMCI) if there was no impairment in memory. A diagnosis of dementia was made according to the Diagnostic and Statistical Manual of Mental Disorders, 4th edition.

### MRI Acquisition

MRI was performed at the First Affiliated Hospital of BeiHua University Department of Radiology. All participants underwent MRI on a 1.5-T SIEMENS NOVUS scanner system (Simens, Erlangen, Germany). The MRI protocol included a three-dimensional T1-weighted gradient echo sequence, a T2-weighted sequence, and a fast fluid-attenuated inversion recovery (FLAIR) sequence. The MRI data were checked for compliance with the imaging protocol and then cataloged. Visual assessment of the MTA and WMH analysis was performed at the imaging center.

### Visual Assessment of MTA

Visual assessment of MTA was performed using coronal T1-weighted images with a 5-point visual rating scale (18). The scores ranged from 0 (no atrophy) to 4 (severe atrophy) based on the height of the hippocampal formation and the surrounding cerebrospinal fluid (CSF) spaces. The dichotomized summed score of both medial temporal lobes was used in the analysis, and an MTA score ≥ 1.5 was considered abnormal. The process was performed under the supervision of two neuroimaging physicians. The severity of white matter hyperintensity was analyzed using the Fazekas scale (19), with scores of 1–6 defining the moderate/severe white matter hyperintensity. MRI readings of WMH were obtained on axial FLAIR images. All MRI readings were performed by two neuroradiologists. Prior to the start of the study, all participating neuroradiologists underwent uniform training to clarify the purpose and significance of the study.

### Genotyping

The APOE genotype was determined in all participants using genomic DNA extracted from EDTA anticoagulated blood via polymerase chain reaction (PCR) amplification and HhaI restriction enzyme digestion (19).

### Laboratory Measures

Non-fasting blood samples were collected from consenting patients into evacuated tubes containing EDTA, centrifuged within 10 min, and stored at or below −20°C until analysis. Plasma total homocysteine concentrations were determined by using fluorescence polarization immunoassay technology (IMX System; Abbott Laboratories, Abbott Park, IL). The CV for this assay was 4.4% (normal values) and 2.2% (high values). Serum concentrations of folate and cyanocobalamin (vitamin B12) were measured with a radioimmunoassay kit with the use of 57Co and 125I, respectively (SimulTRAC-SNB; ICN Pharmaceutical, Orangeburg, NY). The within-run CVs for these assays were 7.1% for serum folate and 12.3% for cyanocobalamin.

### Statistical Analysis

The experimental measurement data are expressed as the means ± standard deviations (SDs). The experimental count data were expressed as numbers and percentages. Analysis of variance (ANOVA) was used to compare differences in the neuropsychological data, MTA scores, WMH scores, and APOE-4 genotype among the MCI subtypes. The cumulative frequency distributions of the Hcy and folate concentrations in patients with aMCI and naMCI were compared with those in healthy control subjects by using the Kolmogorov–Smirnov test. Multiple linear regression analyses were used to test the associations among MTA scores and the APOE genotype with Hcy in the MCI subtypes. A linear relationship between the independent and dependent variables was determined by plotting partial regression scatter plots and scatter plots of studentized residuals and predicted values. All analyses were performed using SPSS PASW Statistics for Windows, version 18.0 (SPSS Inc., Released 2009, Chicago, IL, USA).

## Results

### Sample Characteristics and Neuropsychometric Assessments

The demographic characteristics and neuropsychological test performance of the study sample are presented in Table 1. The groups were well-balanced in terms of their demographic and clinical information. Age, sex, and level of education were not significantly different among the aMCI, naMCI and control groups (Table 2). As expected, the MMSE scores were significantly lower in all MCI subjects than in the controls. Regarding the neuropsychological test scores, the group effects were significant in all cognitive domains, with the best performance in the controls and the worst performance in the aMCI subjects. We also examined the differences in the APOE ε4 genotype prevalence between the aMCI and naMCI groups. The prevalence of APOE ε4 in the aMCI groups (42.3%) was more than twice that in the naMCI groups (19.0%).

**Table 1:** Demographic, neuropsychological, and MRI variables.

	**aMCI**	**naMCI**	**Control**
**Demographics**			
Age (years)	69.7 (6.6)	69.2 (5.0)	69.8 (5.7)
Sex (female), *n* (%)	71 (54.6)	30 (37.7)	58 (54.7)
Education (years)	11.6 (3.9)	11.3 (3.2)	11.4 (3.1)
MMSE	25.7 (2.3)	27.1 (1.4)	28.1 (1.0)
APOE4, *n* (%)	55 (42.3)	11 (19.0)	21 (19.8)
Hcy	14.3 (6.8)	12.2 (4.9)	10.3 (3.7)
Folic acid	8.6 (5.1)	11.3 (7.8)	19.2 (7.7)
Vit B12	458.6 (248.4)	488.1 (256.7)	454.6 (187.1)
**Neuropsychological battery**			
RAVLT-IR	22.1 (4.2)	26.7 (3.3)	28.0 (3.6)
RAVLT-DR	6.1 (1.9)	10.9 (1.3)	11.3 (1.4)
CDT	2.5 (0.6)	2.5 (0.6)	2.8 (0.4)
TMT-A	90 (33.8)	85.9 (30.5)	73 (16)
TMT-B	178.6 (50.2)	171.7 (49.5)	115 (25)
BNT	22.8 (3.6)	23.5 (3.4)	25.8 (1.3)
**MRI**			
MTA scores	1.6 (0.9)	1.1 (0.8)	0.98 (0.62)
Moderate/severe WMH, n (%)	96 (73.8)	41 (70.7)	30 (28.3)

*Values are expressed as the means ± standard deviations (SDs) unless stated otherwise. Analysis of variance (ANOVA) and Bonferonni post hoc tests with age, sex, and education as covariates were performed. MRI, magnetic resonance imaging; Moderate/severe WMH, Moderate/severe white matter hyperintensities; MMSE, Mini-Mental State Examination; RAVLT-IR, Rey Auditory Verbal Learning Task (WHO) immediate recall; RAVLT-DR, Rey Auditory Verbal Learning Task (WHO) delayed recall; TMT, Trail Making Test; CDT, clock drawing test; BNT, Boston naming test; MTA, medial temporal lobe atrophy (score 0–4). ApoE4 was considered present if one or both alleles were E4*.

**Table 2:** Comparisons of the clinical characteristics of the three groups.

**Characteristic**	**Overall P**	**Group comparisons**
Age (years)	0.771	—
Sex, *n* (% female)	0.922	—
Education (years)	0.586	—
MMSE	<0.01	1>3>2
AVLT-IR	<0.01	1, 3>2
AVLT-DR	<0.01	1, 3>2
CDT	<0.01	2>3>1
TMT-A	<0.01	2, 3>1
TMT-B	<0.01	2, 3>1
BNT	<0.01	1>2, 3

*aMCI, amnestic MCI: Group 1; naMCI, non-amnestic MCI: Group 2; control: Group 3. “—” indicates not significant. Intergroup differences, Bonferroni test*.

### Homocysteine and Cumulative Frequency Distributions

**Figure 1:**
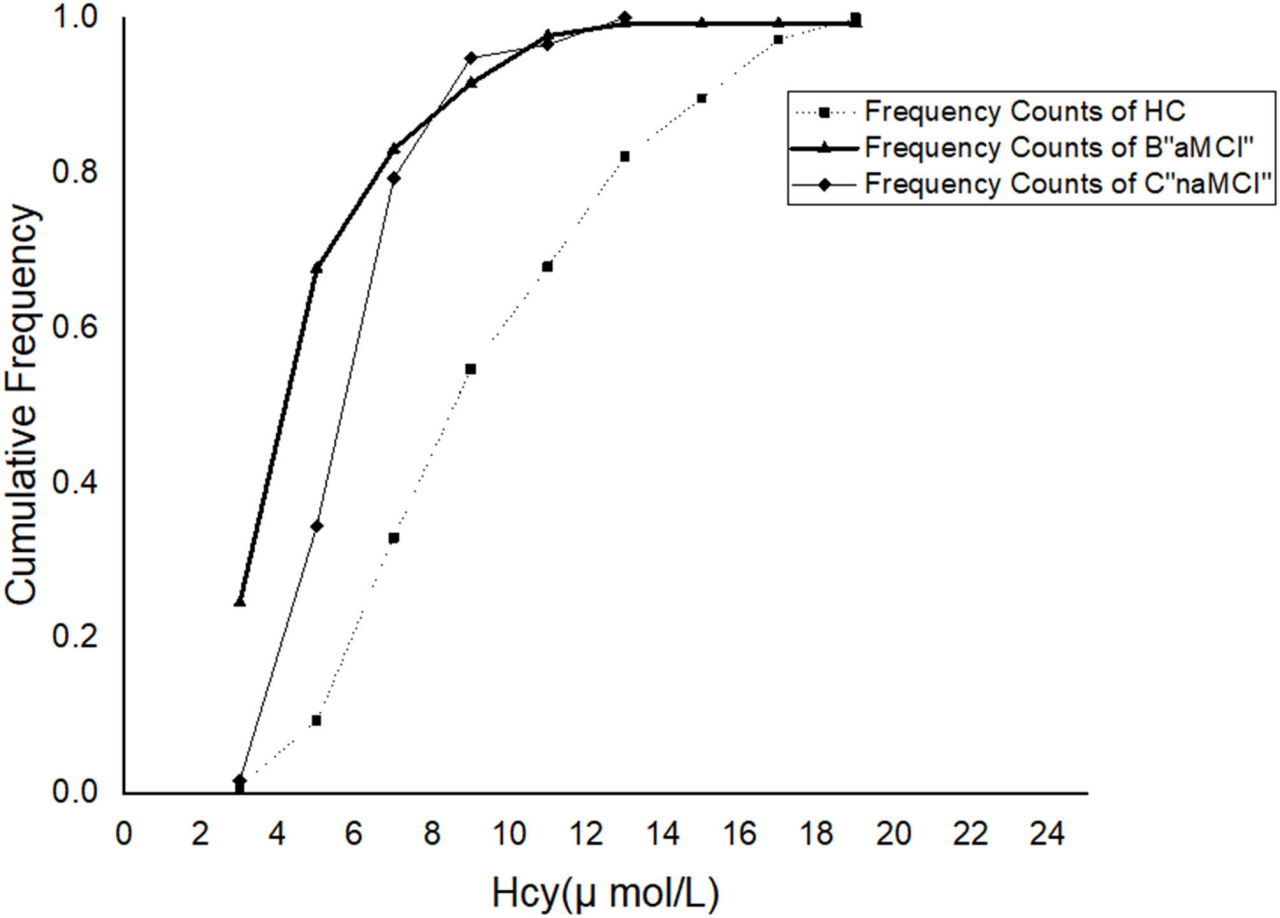
Cumulative frequency distributions of plasma homocysteine (Hcy) levels in the healthy controls and aMCI and aMCI subjects.

Mean serum folate levels were significantly lower in the aMCI and naMCI groups than in the healthy control group (*p* < 0.05). Mean plasma Hcy levels were significantly higher in patients with aMCI and naMCI than they were in healthy controls (*p* < 0.01). The aMCI group had significantly lower mean serum folate levels and higher mean Hcy concentrations than the naMCI group (*p* < 0.05). The cumulative frequency distributions of the Hcy concentrations in the three groups are shown in Figure 1. The cumulative frequency plots show that the accumulation frequency of Hcy was higher in both the aMCI and naMCI groups than in healthy control group. The cumulative frequency plots show a shift in the distribution of the Hcy concentrations to higher values in the patients with aMCI and naMCI compared with healthy controls.

### Homocysteine, APOE4, White Matter Hyperintensities and Medial Temporal Atrophy

As shown in Table 3, increasing baseline hippocampal atrophy severity, which was measured by the visual MTA score, differed among the MCI subtypes (*P* < 0.001). The visual MTA scores in the control group were significantly different than those in the aMCI and aMCI-MD subgroups (both *P* < 0.05) but not the naMCI-MD or naMCI-SD groups (both *P* > 0.05).

**Table 3:** Associations of homocysteine levels with MTA scores of the carriers and non-carriers of APOE ε4 among aMCI patients.

**Variable**	**Non-carriers of APOE** **ε4**				**Carriers of APOE** **ε4**			
	**β-Regression coefficient**	**Standard error**	**Odds ratio (95% CI)**	***p*** **-value**	**β-Regression coefficient**	**Standard error**	**Odds ratio (95% CI)**	***p*** **-value**
Hcy	0.84	0.62	2.32 (1.68–3.21)	<0.001	1.73	0.62	5.66 (1.69–13.01)	0.005
Age	0.20	0.42	1.22 (1.06–1.39)	0.004	0.96	0.42	2.61 (1.14–5.97)	0.023
Serum folate	0.06	0.19	1.06 (0.93–1.21)	0.378	0.36	0.19	1.44 (0.98–2.10)	0.063

To further evaluate the relationship between the visual rating scores and the clinical variables, we performed a multivariate regression analysis in Table 4. Age was an independent influencing factor in all three groups (*P* < 0.05). Following adjustments for age and sex, Hcy and serum folate levels and the APOE4 gene were significantly correlated with MTA scores only in the aMCI group (*P* < 0.05). We also observed a significant association of the presence or absence of moderate/severe WMH with MTA in the naMCI and HC groups (*P* = 0.004 and *P* = 0.029, respectively).

**Table 4:** Comparison of MTA, WMH, and APOE4 across MCI and control groups.

**Group**	**Variable**	**β coefficient**	**Std. Error**	***P***
aMCI	**Hcy**	0.925	0.151	**0.000**
	**Serum folate**	0.117	0.057	**0.039**
	Vitamin B12	−0.002	0.001	0.110
	**Age**	0.265	0.069	**0.000**
	Sex	−0.325	0.539	0.547
	**APOE** **ε4**	−1.750	0.625	**0.005**
	Moderate/severe WMH	−0.846	0.640	0.186
naMCI	Hcy	0.004	0.077	0.955
	Serum folate	0.011	0.042	0.792
	Vitamin B12	0.001	0.001	0.501
	**Age**	0.469	0.114	**0.000**
	Sex	−0.743	0.697	0.286
	APOE ε4	1.199	0.869	0.168
	**Moderate/severe WMH**	−3.848	1.332	**0.004**
HC	Hcy	−0.007	0.062	0.907
	Serum folate	−0.042	0.030	0.166
	Vitamin B12	0.000	0.001	0.891
	**Age**	0.088	0.038	**0.022**
	Sex	0.016	0.407	0.968
	APOE ε4	−0.189	0.507	0.709
	**Moderate/severe WMH**	−1.061	0.487	**0.029**

*aMCI-SD, amnestic single-domain MCI; aMCI-MD, amnestic multiple-domain MCI; naMCI-SD, non-amnestic single-domain MCI; naMCI-MD, non-amnestic multiple-domain MCI. Bold values: significant p-values*.

## Discussion

In this study, detailed neuropsychological testing was employed to identify mutually exclusive subtypes of MCI according to cognitive characteristics. The APOE genotype, level of cognitive impairment, and brain pathological structural damage that underlies each subtype are likely distinct. We determined that the aMCI individuals exhibited more severe MTA than the controls. However, the severity of MTA did not significantly differ between the naMCI individuals and controls. These findings are consistent with previous studies that identified MTA in individuals with aMCI (20–23). There was a widespread distribution of hippocampal and medial temporal lobe atrophy in the aMCI individuals compared with the naMCI individuals. These findings reflect episodic memory impairment and parallel the pathology of early AD (24).

An elevated plasma concentration of the sulfur amino acid Hcy is recognized as an independent risk factor for cardiovascular, peripheral vascular, and cerebrovascular disease (25). Studies have pointed to selective effects of Hcy in specific areas of cognition (26). One explanation may be that Hcy may affect some parts of the brain more than others, and studies have shown that Hcy is associated with high levels of white matter hypersignaling and brain atrophy (27). In agreement with these findings (28–30), we did not find any significant association between the serum vitamin B12 concentrations and MCI.

The cumulative frequency plots showed that the frequency distribution of vitamin B12 in the aMCI group was very similar to the distribution observed in the naMCI group. We identified significantly higher Hcy concentrations in MCI patients than in healthy controls. Additional studies are needed to establish the significance of these associations.

Correlation analyses were subsequently performed to examine the relationships between MTA and memory scores. The severity of MTA was directly associated with episodic memory performance in aMCI patients; however, it was not directly associated with episodic memory performance in the naMCI patients, which is consistent with a recent study (23). Our observation may explain the fact that structural lesions of the hippocampus and middle temporal lobes are strongly associated with episodic memory impairments in aMCI. Our results suggest that the classification of MCI patients with and without memory impairment may identify important heterogeneous characteristics. Thus, our findings are in line with previous work indicating that the MTA scale is useful for identifying prodromal stages of AD and distinguishing patients with AD from individuals with other dementias (21, 31). Our neuroimaging findings indicated that there were no differences in WMH among MCI subtypes, which is similar to the results of Bombois et al. (32). However, our findings are inconsistent with another study that identified increased vascular risk factors, WMH, and a history of stroke in individuals with naMCI (32). Overall, our findings suggest that the pathology of cerebrovascular disease may not be heterogeneous across all MCI subtypes.

In addition, our results demonstrated that APOE-4 alleles were more prevalent in individuals with aMCI than in individuals with naMCI and healthy controls. The MTA score and memory performance were strongly associated with the APOE-4 genotype in aMCI individuals but not in naMCI individuals. Furthermore, the APOE-4 allele influenced the severity of MTA and memory impairment in the aMCI subjects. This effect was not identified in the subjects with naMCI. Thus, the APOE-4 allele may represent a risk factor for aMCI patients. Previous studies have demonstrated that the assessment of MTA using a standardized visual rating scale is quick and easy and can accurately predict progression from MCI to AD (33–35). As previously noted, the memory-impaired subtypes of MCI had the greatest degree of hippocampal atrophy and were associated with an increased APOE-4 allele prevalence, which indicates a likelihood that these individuals will also be at risk for subsequent progression to AD (36–38). Our findings indicated that the combination of MTA and the APOE-4 allele substantially influenced memory performance in the aMCI subtypes. Thus, we suggest that the combination of MTA and APOE-4 genotype may represent a promising risk factor for the prediction of progression from aMCI to dementia.

Despite these promising findings, there are several limitations that should be considered in the interpretation of the results. First, the cross-sectional design limits conclusions regarding the prognostic significance of MTA and the APOE-4 genotype in MCI subtypes. Second, the visual assessment of MTA limits interpretations regarding the distinct causal mechanisms that underlie the different MCI subtypes. Finally, despite the recruitment of 188 subjects with MCI, the number of naMCI subjects was relatively small, which limited subgroup comparisons. Future studies should include a larger sample size and employ a longitudinal approach, as well as a more quantitative MRI analysis, such as the assessment of hippocampal and WMH volumes, which may further our understanding of MCI subtypes.

Based on the findings of the present study, it can be concluded that different neuropsychological subtypes of MCI may involve distinct biological processes, and the mechanisms underlying amnestic and non-amnestic MCI may differ. High levels of Hcy may be associated with a higher risk of aMCI. Furthermore, the combined presence of APOE4 and higher levels of Hcy is associated with aMCI in elderly individuals, while WMH is related to naMCI. Although differences in the underlying etiologies of the MCI subtypes may be prevalent, cerebrovascular diseases are common, and the severity of WMH is similar. Therefore, additional attention should focus on the control of cerebrovascular risk factors among all MCI subtypes.

## Data Availability Statement

The original contributions presented in the study are included in the article/supplementary material, further inquiries can be directed to the corresponding author/s.

## Ethics Statement

The studies involving human participants were reviewed and approved by the Medical Ethics Committee of Capital Medical University. The patients/participants provided their written informed consent to participate in this study.

## Author Contributions

GZ designed the study. JM and L-YM interpreted the data and drafted and revised the manuscript. FM performed experiments and revised the manuscript. All authors contributed to the article and approved the submitted version.

## Funding

This work was supported by the fund project of Jilin Province Department of Education: No. 144, 2011.

## Conflict of Interest

The authors declare that the research was conducted in the absence of any commercial or financial relationships that could be construed as a potential conflict of interest.

## Publisher's Note

All claims expressed in this article are solely those of the authors and do not necessarily represent those of their affiliated organizations, or those of the publisher, the editors and the reviewers. Any product that may be evaluated in this article, or claim that may be made by its manufacturer, is not guaranteed or endorsed by the publisher.
